# Study on Autophagy Death of Alpha TC1 Clone 6 (αTC1-6) Cells Induced by Trametenolic Acid Through PI3K/AKT Pathway

**DOI:** 10.3390/cimb47100871

**Published:** 2025-10-21

**Authors:** Wangyang Ye, Shangling Pan, Hongqi Zhang, Xiaolan Zhang, Junzhi Wang

**Affiliations:** 1 School of Basic Medical Sciences, Guangxi Medical University, Nanning 530021, China; 202110009@sr.gxmu.edu.cn; 2 Third-Grade Pharmacological Laboratory on Traditional Chinese Medicine, State Administration of Traditional Chinese Medicine, College of Medicine and Health Sciences, China Three Gorges University, Yichang 443002, China; 3 Hubei Key Laboratory of Natural Products Research, Development & Hubei Research Center for Bioenzyme Engineering Technology, China Three Gorges University, Yichang 443002, China; 202408600021081@ctgu.edu.cn (X.Z.); wangjunzhi@ctgu.edu.cn (J.W.)

**Keywords:** trametenolic acid B, glucagonoma, αTC1-6 cells, autophagy, PI3K/AKT signaling pathway, FoxO1

## Abstract

Glucagonoma, a rare neuroendocrine tumor, lacks targeted treatment drugs. Excessive secretion of glucagon is the main cause of its clinical syndrome. To explore targeted therapeutic drugs that can inhibit glucagon secretion and tumor proliferation, we investigated the effect of Trametenolic Acid (TA) on mouse pancreatic alpha TC1 clone 6 (αTC1-6) cells and its regulatory role in the PI3K/AKT signaling pathway. Cell viability of αTC1-6 cells was assessed via the MTT assay. Glucagon content in cell culture supernatants was measured using an Enzyme-Linked Immunosorbent Assay (ELISA). Autophagic vacuoles were visualized through Monodansylcadaverine (MDC) staining. The expression of autophagy-related proteins including Atg7, LC3 Ⅱ and PI3K/AKT signaling pathway-related proteins mTOR and FoxO1 were determined by Western blot. The results showed that the proliferation of αTC1-6 cells was significantly inhibited by TA in a dose- and time-dependent manner, and the IC_50_ was 140.71, 26.77 and 1.99 μM after treatment of 12, 24, and 48 h, respectively. The secretion of glucagon was significantly inhibited by TA. The MDC staining results showed that the fluorescent labeled autophagic vesicles in the TA group were increased. The Western blot results showed that the expression of Atg7 and LC3 Ⅱ was promoted by TA in a dose-dependent manner, the phosphorylation of PI3K, AKT, mTOR and FoxO1 was significantly inhibited, and the expression of FoxO1 protein was increased. These results demonstrated that TA can inhibit glucagon secretion, induce autophagy, and suppress cell proliferation in αTC1-6 cells. The mechanism may be associated with the PI3K/AKT signaling pathway.

## 1. Introduction

Glucagonoma (GCGN) is a rare pancreatic endocrine tumor, which originates from the pancreatic islet alpha cells, most of which are malignant [1,2]. The typical clinical manifestation is GCGN syndrome, which is a series of metabolic abnormalities caused by excess glucagon in the blood [3]. Therefore, the adjustment of glucagon secretion and its receptor’s effectiveness is a crucial aspect in the therapeutic strategy [4,5,6,7]. At present, there is a scarcity of targeted drug applications for this condition [8,9]. The identification of therapeutic agents for GCGN, particularly targeted medications that are capable of modulating glucagon secretion and function while simultaneously inducing the demise of GCGN cells, holds immense potential in overcoming the long-standing challenges in GCGN treatment.

**Figure 1:**
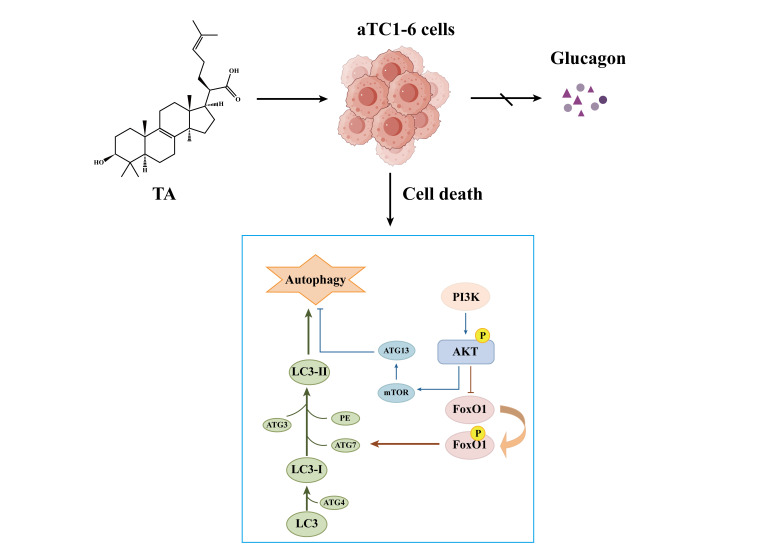
TA can inhibit the proliferation and glucagon secretion of αTC1-6 cells. Specifically, TA is capable of suppressing glucagon secretion in mouse pancreatic αTC1 clone 6 (αTC1-6) cells and inducing their cell death triggered by autophagy activation. This provides a potential approach for the targeted therapy of glucagon-related disorders.

*Poria cocos* (Schw.) Wolf is a traditional Chinese herbal medicine with common effects of promoting diuresis to eliminate dampness, invigorating the spleen, regulating the stomach, calming the mind, and tranquilizing the spirit. It can be used in the treatment of digestive system diseases; in addition, it is applied to the management and regulation of various tumor conditions [10,11,12,13,14,15]. Trametenolic acid (TA) (Figure 1) is a triterpenoid compound with potent antitumor activity isolated from Poria cocos. Previous studies have shown that TA exerts multiple pharmacological effects, including antitumor activity, anti-gastric ulcer effects, neuroprotection, antiviral activity, anti-inflammatory effects, reversal of drug resistance, and enhancement of insulin sensitivity [16,17]. Moreover, by inhibiting PI3K/AKT/mTOR pathway, TA can significantly increase the autophagy level of HepG2/2.2.15 cells and gastric cancer HGC-27 cells, leading to autophagy death of tumor cells [18]. PI3K/AKT is a crucial autophagic signaling pathway that has been extensively validated to be involved in various physiological and pathological processes. It has the capacity to influence tumor cell proliferation and is intimately linked to the development of insulin resistance [19,20,21].

Based on previous studies, TA has been shown to stimulate autophagy in various tumor cell types, which in turn contributes to its antitumor effects. Given that αTC1-6 cells serve as a cell model for glucagonoma (a type of pancreatic neuroendocrine tumor), we hypothesized that the biological effects of TA on αTC1-6 cells might also be mediated through autophagy. Therefore, the present study aims to explore the effects of TA on autophagy in αTC1-6 cells and the PI3K/AKT signaling pathway. This exploration aims to establish a foundation for the development of safe and efficacious drugs that not only modulate glucagon secretion but also eliminate tumor cells (Figure 1), and further contributes for the management of GCGN by presenting novel targeted drugs and therapeutic strategies.

## 2. Materials and Methods

### 2.1. Chemicals and Reagents

Trametenolic acid (TA) was provided by Hubei Key Laboratory of Natural Products Research and Development (China Three Gorges University) with purity over 98% (The structure of TA was shown in Figure 1). Phosphate-buffered saline (PBS, ZA13E01) was obtained from absin Company (Shanghai, China). Penicillin and streptomycin (2149393), Dimethyl sulfoxide (DMSO, 20211011), fetal bovine serum (2222092) were obtained from Gibco Company (Carlsbad, CA, USA). Trypsin (2907B503) and MTT (K0063) were purchased from Sigma Company (St. Louis, MO, USA), MDC kit (20220601) was purchased from Solarbio (Beijing, China). ELISA kit was purchased from Elabscience Biotechnology Co., Ltd. (Wuhan, China), BCA kit was purchased from Beyotime (Shanghai, China).

### 2.2. Cell Culture

Mouse pancreatic αTC1-6 cells (purchased from Shanghai Zeye Biotechnology Co., Ltd., Shanghai, China) were maintained at 37 °C in a humidified atmosphere with 5% CO_2_ in Dulbecco’s Minimum Essential Medium (DMEM) supplemented with 10% fetal bovine serum (FBS). For the experiments, cells of passages No. 3 to 6 were grown to 60–80% confluence and subsequently treated with TA at the specified time points. The TA stock solutions were dissolved in DMSO and then sterilized by passing through filter membranes (Millipore, 0.22 mm pore size). The working solutions were serially diluted in the culture medium to achieve the desired final concentrations.

### 2.3. Cell Viability Assay

αTC1-6 cells were seeded in 96-well plates for 12 h and then treated with TA (5, 10, 20, 40, 60 and 80 μM) continuously for 12 h, 24 h and 48 h. After the incubation, the MTT assay was used to assess the proliferation of TA on the αTC1-6 cells. The experiment was repeated three times to calculate the inhibition rate of TA on cell proliferation.

### 2.4. Detection of Glucagon Secretion

αTC1-6 cells were plated in 24-well culture plates and grown to 50–70% confluence. After treating with TA for 12 h and 24 h, the content of glucagon in the supernatant of cell culture was detected by Elisa kit. Insulin was used as a positive control at a concentration of 5 μM.

### 2.5. Monodansylcadaverine (MDC) Staining

αTC1-6 cells were seeded in a 6-well plates at a density of approximately 1 × 10^5^ cells/well and incubated for 12 h. TA (10, 20, 40 μM) was added and incubated for 12 h. Then, the cells were collected in a 1.5 mL centrifuge tube and centrifuged at 800× *g* for 5 min. The pelleted cells were resuspended in 500 μL 1× wash buffer. In another 1.5 mL centrifuge tube, 90 μL of the cell suspension and 10 μL of MDC staining solution were added, and upon gentle mixing, the mixture was incubated for 30 min in the dark. The mixture was centrifuged at 800× *g* for 5 min. The pelleted cells were washed with 300 μL 1× wash buffer two times and resuspended in 100 μL of collection buffer. Slides were prepared using 5 μL of the cell suspension and observed under a fluorescence microscope to detect autophagy. The intensity of fluorescence was calculated by Image J software (version 1.48).

### 2.6. Western Blot Analysis

The total proteins of αTC1-6 cells were extracted by using RIPA Lysis buffer. The protein concentration was determined by BCA kit. 50 μg of protein in each group was separated by 8% or 12% SDS-PAGE gels and then transferred onto the PVDF membrane. The nitrocellulose membranes with transferred proteins were incubated with primary antibodies against the target proteins (to be verified) and β-actin at 4 °C overnight (The primary antibody required for the experiment is shown in Table 1), and followed by the addition of HRP-labeled secondary antibody and incubation at 37 °C for 1 h. The target protein bands were visualized on X-ray film by ECL coloration. Quantitative analysis was performed using the ImageJ Morphology Analysis System (National Institutes of Health, NIH, Bethesda, Maryland, USA), and the expression levels of target molecules were normalized to that of β-actin.

### 2.7. Statistical Analysis

All experiments were performed in triplets and all data were presented as mean ± SD. Database was set up with SPSS 21.0 software package (SPSS Inc., Chicago, IL, USA), multiple variables were performed by one-way ANOVA, Dunn’s multiple comparison test and Kruskal–Wallis test were used for comparison of variable pairs. *p* < 0.05 was considered statistically significant.

## 3. Results

### 3.1. TA Suppressed the Proliferation of αTC1-6 Cells

**Figure 2:**
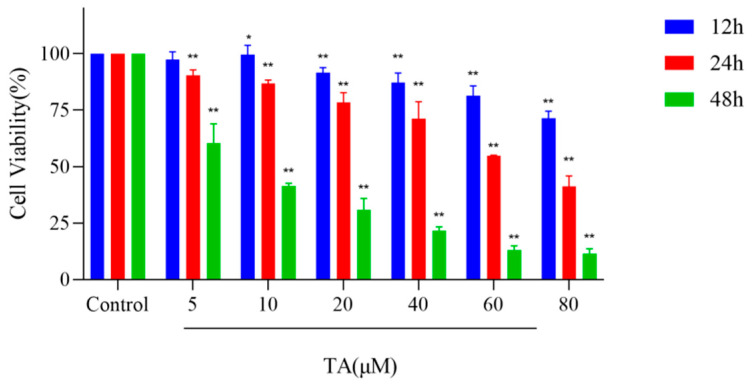
Effect of TA on cell proliferation and cytotoxicity in αTC1-6 cells. The ordinate represents cell inhibition rate (%). The results are expressed as mean ± SD (*n* = 3). * *p* < 0.05 and ** *p* < 0.01 indicate significant differences compared to the control group.

After treatment with TA for 12, 24, and 48 h, the proliferation of αTC1-6 cells was significantly inhibited in a dose- and time-dependent manner (*p* < 0.05), with IC50 values of 140.71 μM, 26.77 μM, and 1.99 μM, respectively. These results indicated that TA had a good inhibitory effect on proliferation of αTC1-6 cells (Figure 2).

### 3.2. TA Inhibited Glucagon Secretion in αTC1-6 Cells

**Figure 3:**
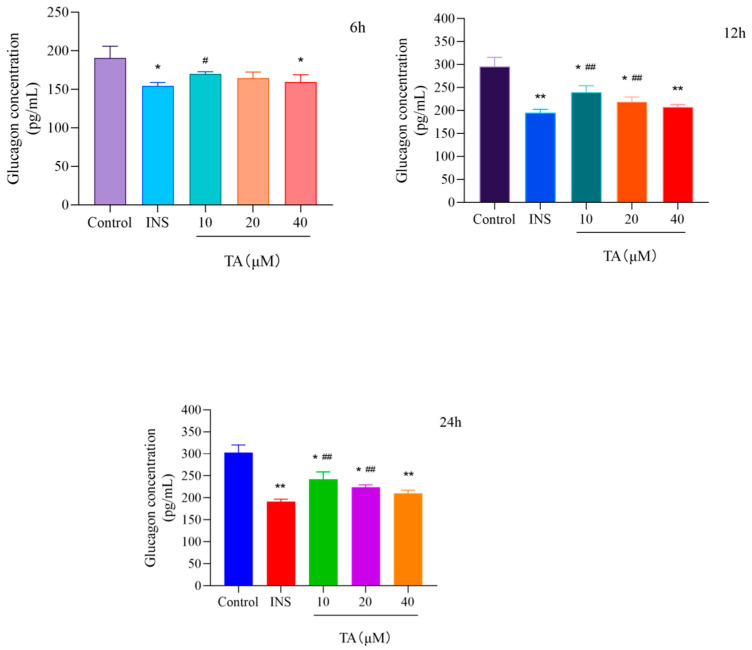
Effects of TA treatment for 6, 12 and 24 h on glucagon secretion in αTC1-6 cells. The results are expressed as mean ± SD performed on (n = 3). * *p* < 0.05 and ** *p* < 0.01 show a significant difference compared to the control group, ^#^ *p* < 0.05 and ^##^ *p* < 0.01 show a significant difference compared to the INS group.

**Figure 4:**
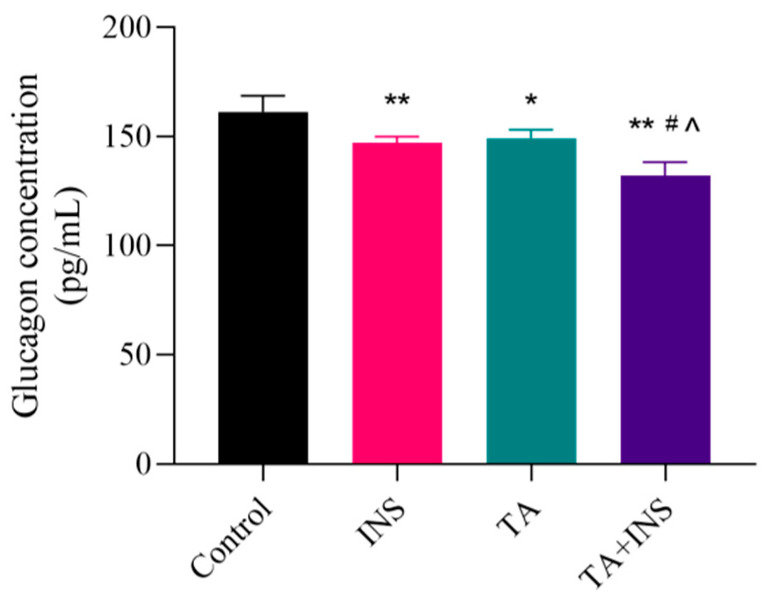
Effects of TA combined with Insulin on glucagon secretion in αTC1-6 cells for 12 h. The results are expressed as mean ± SD performed on *n* = 3. * *p* < 0.05 and ** *p* < 0.01 show a significant difference compared to the control group, ^#^ *p* < 0.05 show a significant difference compared to the INS group, ^^^ *p* < 0.05 show a significant difference compared to the TA group.

αTC1-6 cells treated with 5 μM insulin (INS) were selected as positive control group. After 12 h of treatment with TA, glucagon secretion was inhibited dose-dependently as compared with the control group, and the results at 24 h were not significantly different from those at 12 h (Figure 3). No significant difference was observed between high dose (40 μM) and positive control group (INS group), indicating that higher dose of TA had a similar inhibitory effect on glucagon secretion as insulin. When TA (10 μM) was combined with insulin, the inhibitory effect on glucagon secretion was greater than that of each drug administered individually (Figure 3 and Figure 4).

### 3.3. Effect of TA on the Autophagy of αTC1-6 Cells

**Figure 5:**
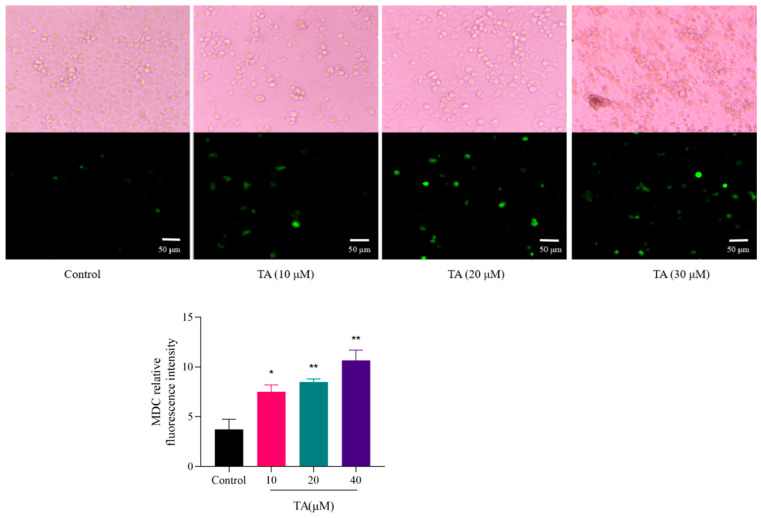
MDC-labeled autophagic vacuoles in αTC1-6 cells (×200). Bright spot fluorescence represents autophagy vacuoles, and the higher the fluorescence intensity, the higher the autophagy level. The relative fluorescence intensity is shown in the histogram. * *p* < 0.05 and ** *p* < 0.01 show a significant difference compared to the control group.

To investigate the effect of TA on autophagy, autophagosomes in αTC1-6 cells were detected by MDC staining where MDC bound to autophagic lysosomes and emitted green fluorescence. As shown in Figure 5, with the increase in TA concentration, the number of green fluorescent vacuoles and thus the fluorescence intensity in the TA-treated group were significantly higher than those in the control group, demonstrating that TA promotes autophagic death in αTC1-6 cells.

### 3.4. Effect of TA on the Expression of Autophagy-Related Proteins in αTC1-6 Cells

**Figure 6:**
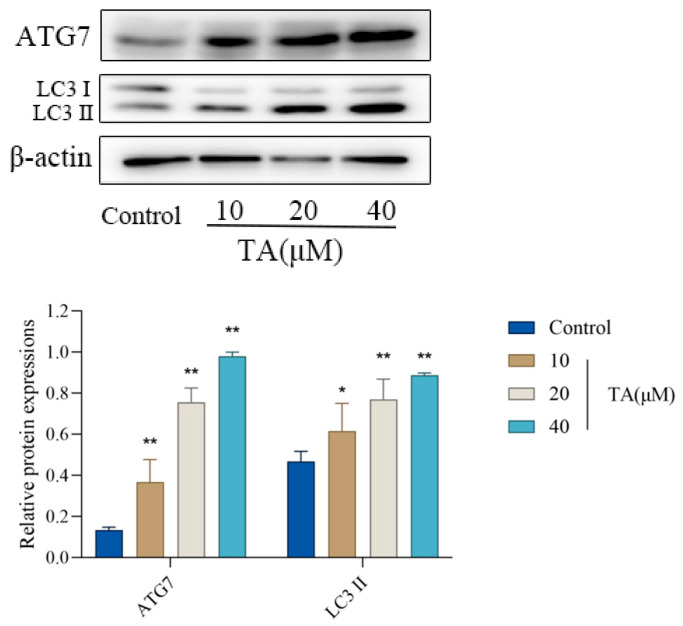

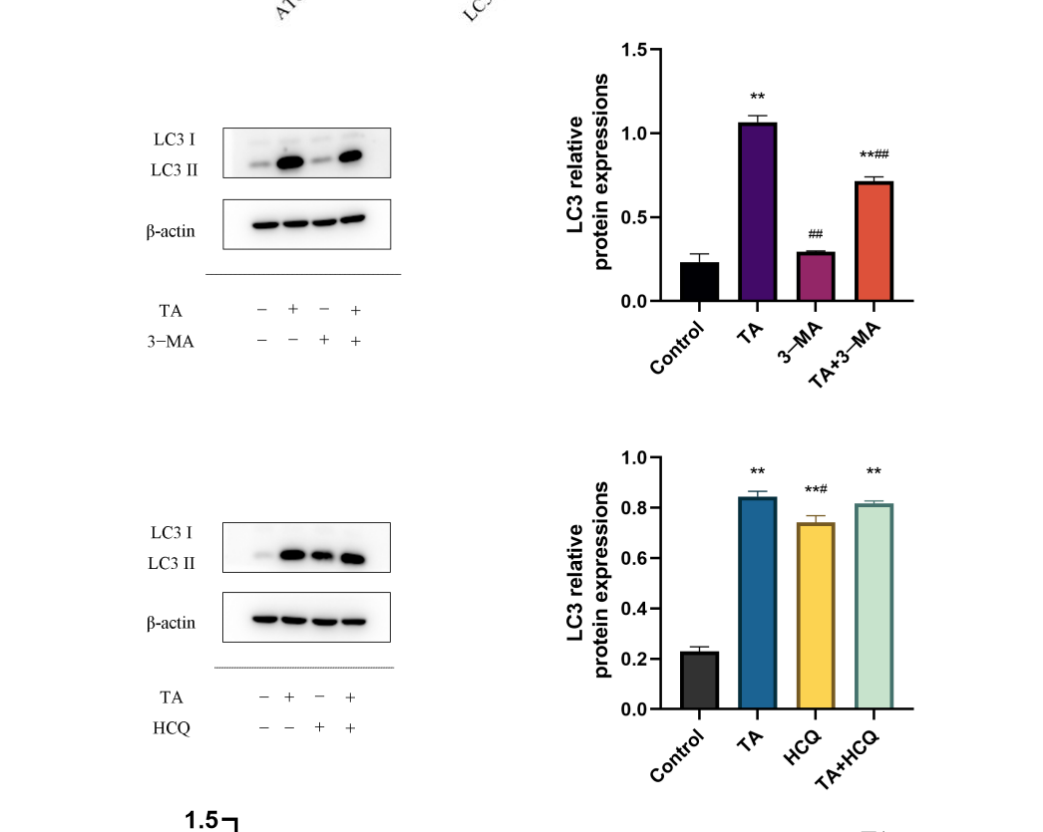

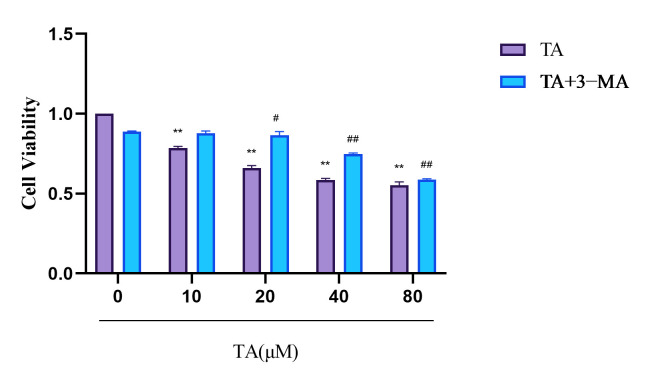

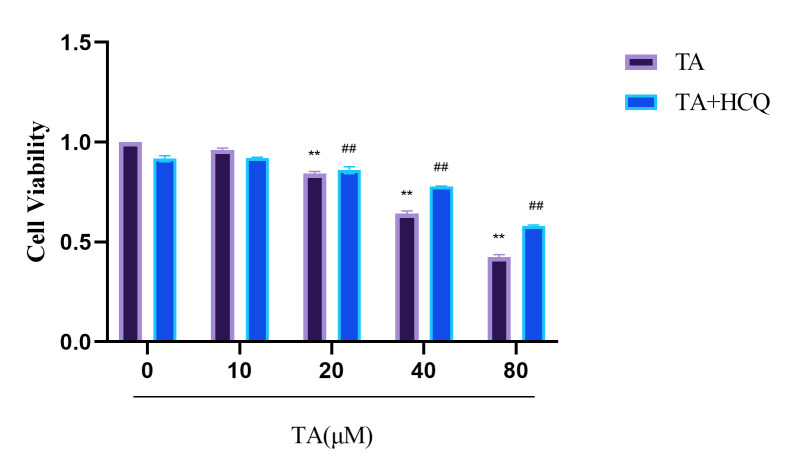
Effects of TA on the expression of ATG7 and LC3Ⅱ proteins in αTC1-6 cells, as well as LC3Ⅱ protein expression and cell viability following treatment with 3-MA or HCQ. Data are presented as mean ± SD (*n* = 3). * *p* < 0.05 and ** *p* < 0.01 indicate significant differences compared to the control group; ^#^ *p* < 0.05 and ^##^ *p* < 0.01 indicate significant differences compared to the TA group.

As shown in Figure 6, compared with control group, the level of autophagy-associated proteins ATG7 and LC3II increased significantly in a concentration-dependent manner after being treated with TA, indicating that TA inhibited the proliferation of α TC1-6 cells by enhancing autophagy activity.

After the addition of 3-methyladenine (3-MA), compared with the TA group, the relative expression level of LC3 protein in the 3-MA group decreased significantly (^##^ *p* < 0.001); while compared with the TA group, the relative expression level of LC3 protein in the TA + 3-MA group decreased significantly (^##^ *p* < 0.001), and was slightly higher than that in the group treated with 3-MA alone. After the addition of hydroxychloroquine (HCQ), compared with the TA group, the relative expression level of LC3 protein in the HCQ group decreased (^#^ *p* < 0.05); while there was no significant change in the relative expression level of LC3 protein in the TA + HCQ group compared with the TA group. This indicates that TA may play a role in promoting the autophagy process in the subsequent lysosomal degradation step. In addition, TA can counteract the inhibition of 3-MA to a certain extent, and the overall situation shows that the initiation of autophagy is significantly inhibited.

### 3.5. Effect of TA on PI3K/AKT Pathway Related Protein Expression in αTC1-6 Cells

**Figure 7:**
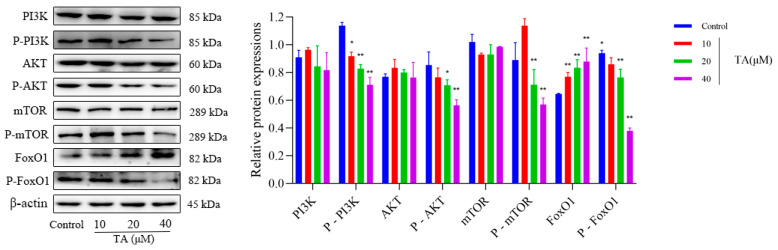
Effect of TA on the related proteins in PI3K/AKT pathway. The results are expressed as mean ± SD performed on (n = 3). * *p* < 0.05 and ** *p* < 0.01 show a significant difference compared to the control group.

As shown in Figure 7, TA significantly inhibited the expression of phosphorylated PI3K, AKT, mTOR and FoxO1 protein while significantly increased the expression of FoxO1 protein which suggested that TA stimulated autophagy in αTC1-6 cells by inhibiting PI3K/AKT signaling pathway through the regulation of both PI3K/AKT/mTOR and PI3K/AKT/FoxO1 axes.

## 4. Discussion

Glucagonoma, a slow-growing pancreatic neuroendocrine tumor, autonomously secretes excessive amounts of glucagon due to the lack of regulation by the body’s negative feedback mechanisms, leading to glucagonoma syndrome [19]. Clinically, the primary manifestations are necrolytic migratory erythema (NME), elevated blood glucose levels, anemia, and chronic inflammation, particularly evident in areas like the corners of the mouth, lips, and tongue [20,21,22]. At present, surgery remains the viable treatment option for the cure of GCGN [2,23]. In the event that the tumor cannot be fully excised or resected, additional chemotherapy and medical interventions are still deemed necessary [24].

Endocrine therapy is of great significance for GCGN [25], as it reduces plasma glucagon levels by inhibiting the secretion of glucagon, rapidly alleviating symptoms [26,27,28]. However, these drugs cannot directly kill tumor cells, and whether they can inhibit tumor growth remains controversial [9]. To achieve the dual purposes of inhibiting glucagon secretion and killing tumors, endocrine therapy is often combined with chemotherapeutic agents. Nevertheless, further research is needed to confirm the efficacy, especially since studies have shown that GCGN is resistant to chemotherapy and have poor systemic chemotherapy efficacy, which requires high attention. Targeted drugs have brought new hope for the treatment of GCGN, but currently there is a lack of specific targeted drugs for the treatment of glucagonoma, and the long-term application of targeted drugs is highly likely to lead to drug resistance. Due to the small size and non-specific symptoms of early stage glucagon tumors, they are prone to misdiagnosis. Therefore, when diagnosed, they are mostly in the late stage and have already metastasized [29]. The common metastatic sites are the liver, lymph nodes, and mesentery/greater omentum/peritoneum [3]. Many patients eventually die from complications, the most common of which are thrombosis [30], sepsis, and gastrointestinal bleeding [31]. Hence, it is of great practical importance to investigate and develop targeted drugs for the treatment of GCGN. The present study reveals that TA effectively inhibits glucagon secretion and concurrently eradicates tumor cells, providing a novel approach for early intervention, treatment, and postoperative reinforcement of GCGN.

The mechanism of TA inhibiting glucagon secretion and inducing autophagic death in GCGN cells is closely related to the PI3K/AKT signaling pathway. FoxO1 and mTOR are downstream genes of the PI3K/AKT pathway [32]. FoxO1 is a crucial autophagy-inducing factor that exhibits abnormal expression in diverse tumor cells, encompassing pancreatic cancer [33,34]. The FoxO1 activity is primarily regulated by its own level of protein phosphorylation, with the PI3K/AKT signaling pathway serving as the direct upstream regulator of FoxO1 phosphorylation [35]. By inhibiting the PI3K/AKT signaling pathway, TA weakens the phosphorylation of multiple residues in FoxO1, thereby enhancing the transcriptional activity of FoxO1 [27,28,29,30,31,32,33,34,35,36]. FoxO1 promotes the binding of phosphatidylethanolamine (PE) to LC3-I by directly interacting with the cytoplasmic autophagy-related protein ATG7, forming LC3-II, and subsequently facilitates the binding of LC3-II to the autophagosome membrane, thereby enhancing autophagy [37,38,39]. Studies have shown that autophagy plays an important role in the development of insulin resistance [40,41]. Furthermore, after the addition of 3-methyladenine (3-MA), an inhibitor of autophagosome initiation (also a class III PI3K inhibitor), the level of LC3-II induced by trametenolic acid (TA) was significantly reduced. In contrast, hydroxychloroquine (HCQ), an inhibitor of autophagosome-lysosome fusion, did not further increase the LC3-II level in the TA-treated group. These results suggest that TA exerts its effect primarily by promoting autophagosome initiation rather than blocking autophagic degradation. Both impaired autophagy and inhibition of ATG7 can induce disruption of the insulin pathway and insulin tolerance, meaning that decreased autophagy activity can lead to insulin resistance [42]. In the present study, the results showed that TA can reduce glucagon levels in αTC1-6 cells, which is probably related to that TA improved autophagy activity and insulin resistance by inhibiting PI3K/AKT signaling pathway. Furthermore, previous studies have affirmed that TA has hypoglycemic activity, which is related to the improvement of insulin sensitivity. Therefore, TA directly inhibits glucagon secretion and improves insulin resistance, and indirectly regulates glucagon secretion and function, both of which are beneficial to the treatment of GCGN.

mTOR, another important downstream effector of the PI3K/AKT signaling pathway, is involved in a variety of functions such as cell growth and proliferation and maintenance of energy homeostasis [43]. mTOR is likely to be the core regulator of autophagy [44]. The mTOR signaling is complexly regulated within cells and is modulated by multiple signaling pathways [45,46,47]. PI3K/AKT signaling pathway, which is upstream of mTOR, can activate autophagy by inhibiting mTOR [15]. The previous study showed that TA induced autophagic death of various tumor cells including hepatocellular carcinoma HepG2/2.2.15 and gastric cancer HGC-27 cells by inhibiting mTOR through PI3K/AKT signaling pathway [48]. In present study, the results showed that TA induced autophagic death in glucagonoma αTC1-6 cells, which was also associated with inhibition of mTOR, demonstrating that TA can inhibit PI3K/ AKT signaling pathway, and then activate autophagy by affecting FoxO1, mTOR and other downstream signals. AKT may be the core target affected by TA. Notably, AKT may serve as the core target of TA. Consistent with our findings, TA may promote the initiation of autophagy, which in turn inhibits the PI3K/AKT signaling pathway—this regulatory cascade is supported by our observation that TA reduces LC3-II levels following 3-MA treatment (an autophagy initiation inhibitor) and suppresses the phosphorylation of AKT.

AKT also plays an important role in the development of malignant tumors, participating in the regulation of various tumor cell signaling pathways, promoting cell proliferation, inhibiting apoptosis, regulating autophagy, and affecting the inflammatory and tumor micro-environment [49,50]. Precisely regulating AKT activity can provide new approaches for clinical treatment of inflammation-related diseases, tumors, and overcoming resistance in malignant tumors. Therefore, targeting the AKT signaling pathway may be an effective anti-tumor prevention and treatment measure [51]. Currently, inhibitors targeting the PI3K/AKT signaling pathway have begun to be developed, such as the AKT inhibitor Perifosine [52,53], which is preparing to enter phase III clinical trials, and the mTOR inhibitor Temsirolimus [54,55], which has been applied in clinical practice, However, there is an issue of increased adverse reactions and the effects are still not satisfactory [56].

Therefore, extensive basic and clinical research is needed in the future to develop potent AKT inhibitors for tumor therapy, which will ultimately contribute to the development of effective clinical treatments for tumors. TA possesses the capability of inhibiting AKT, effectively suppressing the PI3K/AKT signaling pathway, triggering autophagy-mediated death in diverse tumor cells, and reversing acquired drug resistance, including but not limited to paclitaxel and sorafenib. TA is a prevalent active constituent of many antitumor drugs such as *Poria cocos* and *Inonotus obliquus*, providing the basis for the discovery of secure and potent inhibitors targeting the PI3K/AKT signaling pathway. However, the function of autophagy in tumor cells is dual—it can either protect cells under stress conditions, helping them resist damage and maintain survival, or be excessively activated under the regulation of specific signals, ultimately leading to cell death. Both its specific regulatory mechanisms and modulation methods require us to conduct more in-depth exploration.

*Poria cocos* is extensively utilized in the treatment of digestive system tumors, and its efficacy has been fully affirmed [57,58,59]. It can be used for both food and medicine, with long-term application and safety assurance [60,61]. The present study provides a basis for further research of *Poria cocos* on anti-tumor components and mechanisms [62,63,64,65].

## 5. Conclusions

Trametenolic acid (TA) exerts dual therapeutic effects in αTC1-6 cells (a cell model of glucagonoma, GCGN): it inhibits glucagon secretion and induces autophagic cell death. These effects are mediated, at least in part, by the inhibition of the PI3K/AKT signaling pathway (via suppressing AKT phosphorylation and regulating downstream FoxO1/mTOR signaling), supporting TA as a potential targeted agent for GCGN treatment.

## Figures and Tables

**Table 1:** Information of primary and secondary antibodies used for Western blot analysis.

Name	Molecular Weight (kDa)	Dilution Rate	Art. No.	Company
LC3A/B	14/16	1:1000	12741S	Cell Signaling Technology
ATG7	78	1:1000	8558S	Cell Signaling Technology
β-actin	45	1:1000	5125S	Cell Signaling Technology
PI3K	85	1:1000	4249S	Cell Signaling Technology
p-PI3K	85	1:1000	17366S	Cell Signaling Technology
AKT	60	1:1000	4691S	Cell Signaling Technology
p-AKT	60	1:1000	4060S	Cell Signaling Technology
mTOR	289	1:1000	2983S	Cell Signaling Technology
p-mTOR	289	1:1000	5536S	Cell Signaling Technology
FoxO1	82	1:1000	2880S	Cell Signaling Technology
p-FoxO1	82	1:1000	9461S	Cell Signaling Technology
Anti-rabbit IgG	-	1:5000	7074S	Cell Signaling Technology

## Data Availability

The original contributions presented in this study are included in the article/Supplementary Material. Further inquiries can be directed to the corresponding author(s).

## Supplementary Materials

The following supporting information can be downloaded at: https://www.mdpi.com/article/10.3390/cimb47100871/s1

## Abbreviations

TA	Trametenolic Acid
αTC1-6	alpha TC1 clone 6
MDC	Monodansylcadaverine
GCGN	Glucagonoma
INS	Insulin
