# Supplementary material for: Study on Autophagy Death of Alpha TC1 Clone 6 (αTC1-6) Cells Induced by Trametenolic Acid Through PI3K/AKT Pathway
Source: Curr Issues Mol Biol. 2025 Oct 21;47(10):871. doi: 10.3390/cimb47100871 (PMC12564410; doi:10.3390/cimb47100871)
Supplement: Supplementary file 1 [file cimb-47-00871-s001.zip › cimb-3901399-supplementary.pdf]

MDC

Control

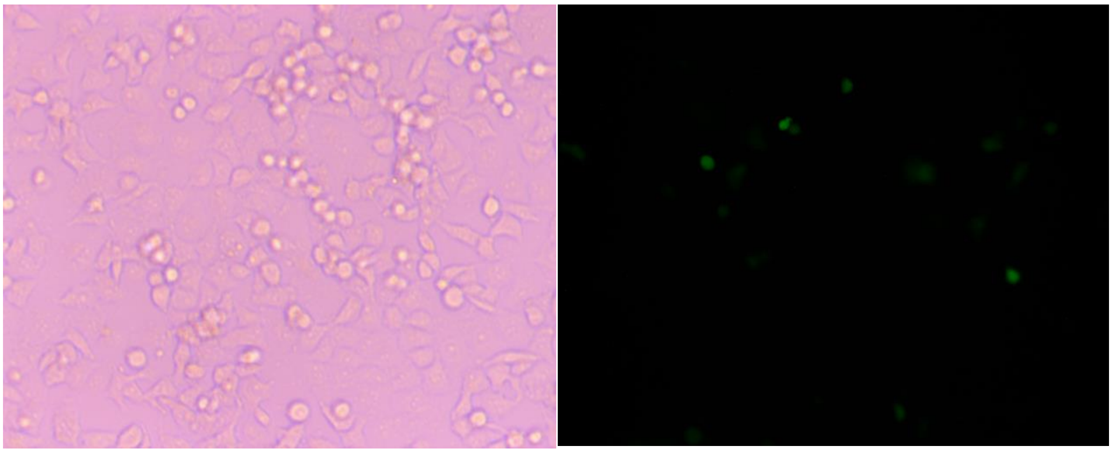

TAB-10 μM

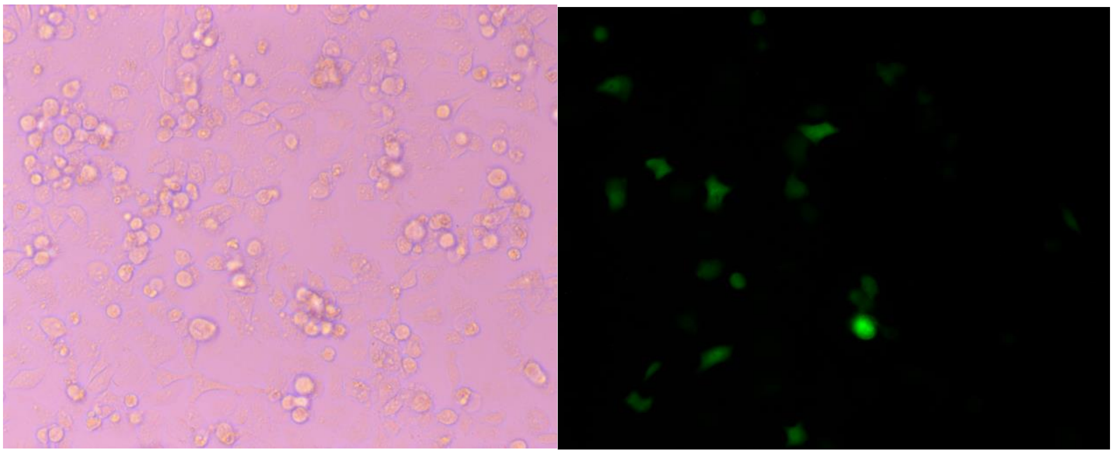

TAB-20 μM

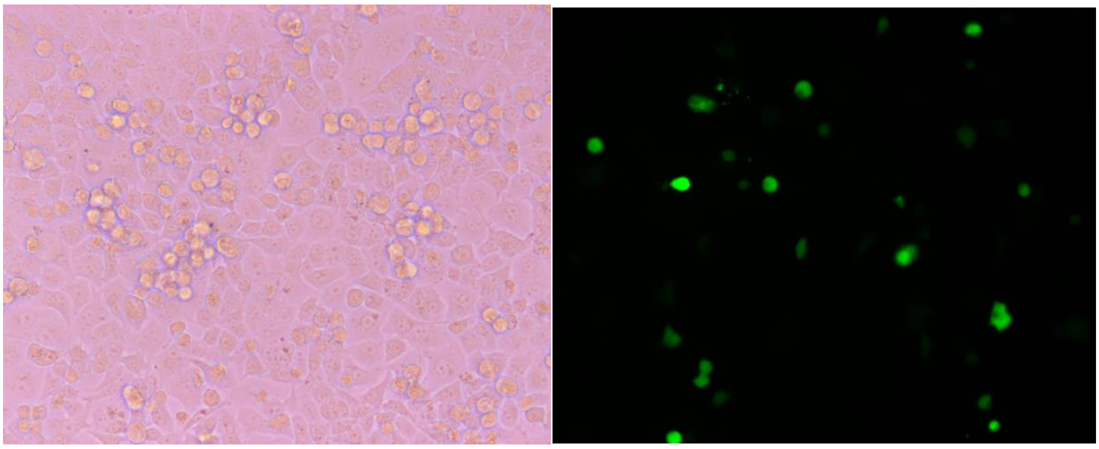

TAB- 40 μM

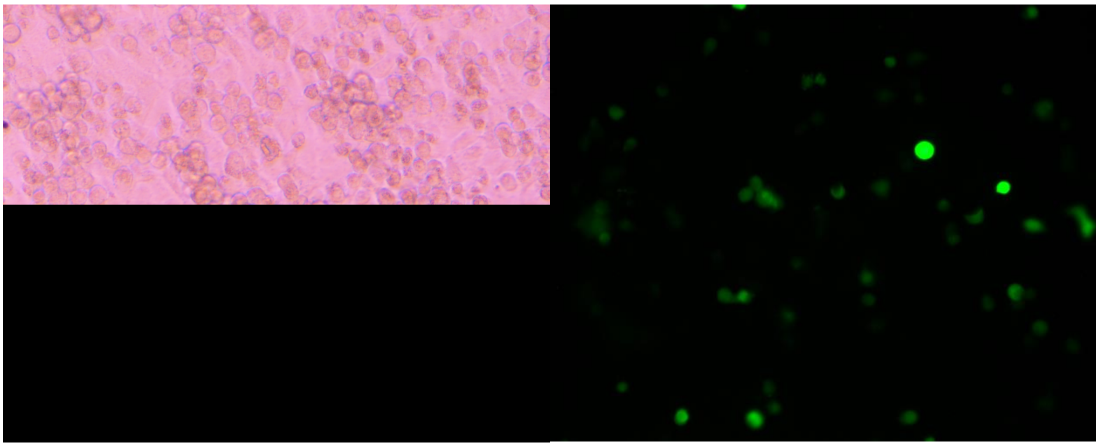

Western blot-1

β-actin

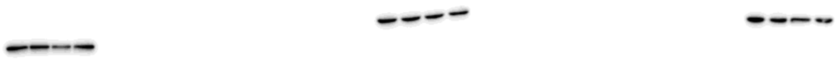

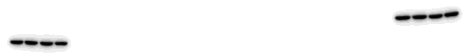

ATG7

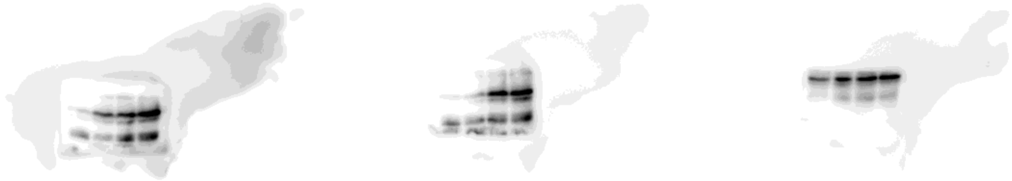

LC3

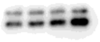

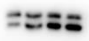

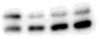

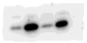

Western blot-2

β-actin

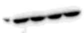

PI3K

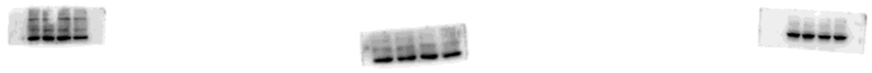

1. PI3K

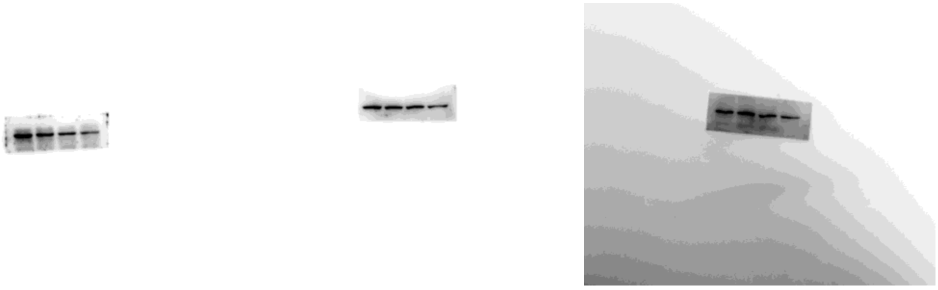

AKT

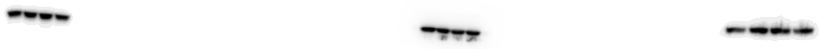

p-AKT

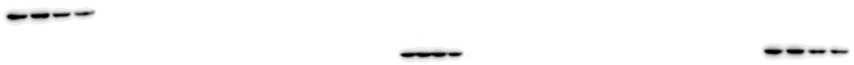

mTOR

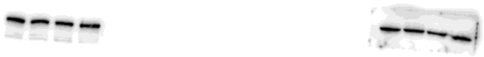

p-MTOR

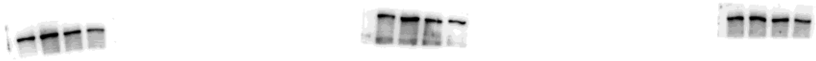

FoxO1

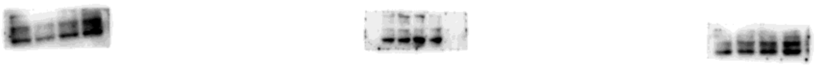

p-FoxO1

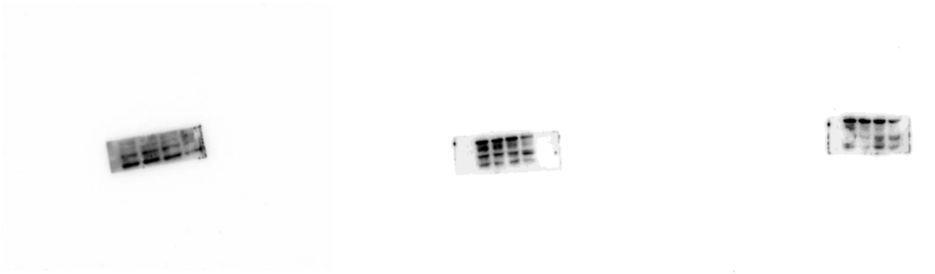

I. Pre-Experimental Preparation

1. Reagent Preparation (Aseptic Operation Throughout to Avoid Contamination)

(1) Stock Solutions and Working Solutions of Core Drugs

TA Stock Solution: Accurately weigh TA powder, dissolve it in cell culture-grade DMSO to prepare a 100 mM stock solution. Vortex to mix well, then filter-sterilize through a 0.22 μm filter membrane. Aliquot into sterile EP tubes and store at -20°C in the dark (single-use aliquots are recommended to avoid repeated freeze–thaw cycles).

3-MA Stock Solution: Weigh 3-MA powder, dissolve it in sterile PBS (pH 7.4) to prepare a 100 mM stock solution. Sonicate to assist dissolution if precipitation occurs, then filter-sterilize through a 0.22 μm filter membrane. Store at 4°C in the dark (valid for 1 week).

HCQ Stock Solution: Weigh HCQ powder, dissolve it in sterile PBS (pH 7.4) to prepare a 10 mM stock solution. Vortex to mix well, then filter-sterilize through a 0.22 μm filter membrane. Store at 4°C in the dark (valid for 1 week).

(2) Dilution of Experimental Working Solutions (Diluted with αTC1-6 Cell-Specific Complete Medium Containing 10% FBS, e.g., DMEM/F12)

(3) Other Basic Reagents

MTT Solution: Prepare a 5 mg/mL solution by dissolving MTT in sterile PBS (pH 7.4). Filter-sterilize through a 0.22 μm filter membrane and store at 4°C in the dark (valid for 2 weeks; re-prepare if precipitation occurs).

Digestion and Washing Reagents: 0.25% Trypsin-EDTA (containing 0.02% EDTA), sterile PBS (pH 7.4), complete medium (containing 10% fetal bovine serum + 1% penicillin-streptomycin double antibody).

Solubilization Reagent: Cell culture-grade DMSO (store at room temperature in the dark; wear gloves during operation to avoid skin contact).

2. Equipment Preparation and Calibration

96-Well Cell Culture Plates (Flat-Bottomed, e.g., Corning Brand): Wipe the surface with 75% ethanol in advance, then place in a biosafety cabinet for UV sterilization for 30 minutes.

Cell Operation Equipment: Pipettes (10 μL, 100 μL, 1000 μL) with corresponding enzyme-free sterile tips, hemocytometer, 0.4% trypan blue staining solution, inverted microscope.

Culture and Detection Equipment: CO_2 incubator (calibrated in advance to 37°C ± 0.5°C, 5% CO_2 ± 0.5%, ensuring saturated humidity), microplate reader (preheated for 30 minutes, calibrated for detection wavelength of 490 nm and reference wavelength of 630 nm), decolorizing shaker (adjustable speed: 0-200 rpm).

II. Cell Preparation (Key: Ensure Cells Are in Logarithmic Growth Phase)

Cell Thawing and Passage

Retrieve cryopreserved αTC1-6 cells, rapidly thaw in a 37°C water bath, then add 5 mL of complete medium. Centrifuge at 1000 rpm for 5 minutes, discard the supernatant, resuspend the cells in complete medium, and seed into a culture dish. Incubate in a 37°C, 5% CO_2 incubator. Passage when cell confluency reaches 70%-80% (limit passage number to 20-30 to avoid cell senescence).

Preparation of Single-Cell Suspension

Discard the old medium in the culture dish, gently wash the cells twice with sterile PBS (to remove residual serum and avoid affecting digestion).

Add an appropriate amount of 0.25% Trypsin-EDTA (just enough to cover the cell layer), incubate at 37°C for 1-2 minutes. Observe under an inverted microscope until cell gaps widen and cells become rounded (avoid over-digestion to prevent cell lysis).

Immediately add twice the volume of complete medium to terminate digestion. Gently pipette the cells with a 1 mL pipette (in a "zig-zag" pattern along the dish wall; avoid excessive force) to prepare a single-cell suspension.

Cell Counting and Concentration Adjustment

Mix 10 μL of cell suspension with 10 μL of trypan blue staining solution, add to a hemocytometer, and count viable cells under an inverted microscope (viable cells are transparent and exclude trypan blue; dead cells appear blue). The viability rate must be > 95%.

Adjust the cell concentration to 5×10^4 cells/mL with complete medium (ensuring each 100 μL of cell suspension contains 5×10^3 cells to meet the seeding volume per well).

III. Experimental Operation Steps (Divided into "Pretreatment Groups" and "Direct Treatment Groups"; See Notes for 96-Well Plate Layout)

1. 96-Well Plate Layout Design (Avoid Edge Effects and Improve Repeatability)

Edge Well Treatment: Add only complete medium (blank control for background correction) to wells in Column 1, Column 12, Row A, and Row H; do not seed cells.

Middle Well Grouping: Arrange "treatment time" (12 h, 24 h, 48 h) horizontally and "drug concentration" (from low to high) vertically. Set 3 replicate wells for each group (to reduce random errors). See the example below for specific layout:

Layout for Combination Groups: Use a separate 96-well plate (or reserve an area in the same plate). Follow the same layout logic, replacing the "TA Treatment Group" with the "TA + 3-MA Combination Group" and "TA + HCQ Combination Group".

2. Cell Seeding and Pretreatment (3-MA/HCQ Require Preincubation to Block Autophagic Pathways)

Cell Seeding: Add 100 μL of the adjusted αTC1-6 cell suspension (5×10^3 cells) to each well. Add 100 μL of complete medium to blank control wells. Gently tap the edge of the 96-well plate to distribute cells evenly (avoid clumping), then incubate in a 37°C, 5% CO_2 incubator for 12 hours. Confirm under an inverted microscope that the cell adherence rate is > 90% (incomplete adherence will lead to uneven drug action).

3-MA/HCQ Pretreatment (for "3-MA Pretreatment Group", "TA + 3-MA Combination Group", "HCQ Pretreatment Group", "TA + HCQ Combination Group"):

Aspirate the old medium from the wells of the above groups using a 100 μL pipette (operate slowly along the well wall to avoid damaging adherent cells).

Add 100 μL of 3-MA working solution (final concentration: 1 mM) or HCQ working solution (final concentration: 5 μM) to the respective wells. Incubate in the incubator for 2 hours (refer to the standard pretreatment time for autophagy inhibitors to ensure effective pathway blocking).

Direct Treatment Groups (TA Alone Group, Solvent Control Group, Blank Control Group): No pretreatment; directly aspirate the old medium and add the corresponding treatment solution.

3. Drug Treatment and Incubation (Precise Control by Time Point)

Drug Addition After Pretreatment: After 2 hours of 3-MA/HCQ pretreatment, aspirate the pretreatment solution. Add 100 μL of complete medium to the "3-MA Pretreatment Group" and "HCQ Pretreatment Group"; add 100 μL of TA + 3-MA/HCQ combination working solution (at the corresponding concentration) to the "TA + 3-MA Combination Group" and "TA + HCQ Combination Group".

Direct Drug Addition: Add 100 μL of TA working solution (at the corresponding concentration) to the "TA Alone Group"; add 100 μL of complete medium containing 0.1% DMSO to the "Solvent Control Group"; retain 100 μL of complete medium in the "Blank Control Group".

Grouped Incubation: Place the 96-well plate in the incubator and incubate for 12 h, 24 h, and 48 h respectively (retrieve at the preset time points to avoid over-incubation). Observe cell morphology daily under an inverted microscope (e.g., whether cells shrink or float) to preliminarily assess drug toxicity.

4. MTT Detection (Key Step: Ensure Complete Solubilization of Formazan Crystals)

MTT Addition: Add 20 μL of MTT solution (5 mg/mL) to each well 4 hours before the end of incubation for each time point. Gently shake the plate to mix MTT with the medium thoroughly (avoid generating air bubbles), then return to the incubator and incubate for 4 hours in the dark (MTT is light-sensitive; avoid opening the incubator door during incubation).

Supernatant Aspiration: After incubation, carefully aspirate the supernatant from each well using a 100 μL pipette (for adherent cells, aspirate slowly along the well wall; for suspension cells, centrifuge at 1000 rpm for 5 minutes first, then aspirate the supernatant to avoid removing the blue-purple formazan crystals at the bottom of the wells).

Crystal Solubilization: Add 150 μL of DMSO to each well, seal the edge of the 96-well plate with parafilm (to prevent DMSO volatilization), and place on a decolorizing shaker. Shake at a low speed (50-80 rpm) for 10 minutes at room temperature in the dark. Confirm under an inverted microscope that formazan crystals are completely solubilized (no obvious particles; the solution is uniformly blue-purple).

5. Absorbance Measurement (Eliminate Background Interference to Ensure Data Accuracy)

Microplate Reader Setup: Set the detection wavelength to 490 nm (maximum absorption peak of formazan crystals) and the reference wavelength to 630 nm (to correct for non-specific absorption from medium, DMSO, and plate material). Enable the "blank correction" function.

Sample Detection: Gently tap the 96-well plate on the desktop to remove air bubbles (bubbles will cause low OD values), immediately place it in the microplate reader, start the detection program, and record the "corrected OD value" for each well (i.e., OD value at 490 nm minus OD value at 630 nm).

IV. Data Processing and Analysis (Standardized Process to Improve Result Reliability)

1. Basic Data Collation

Calculate the average OD value of 3 replicate wells in each group. Exclude outliers (if the OD value of a single replicate well differs by > 20% from the average, verify whether it is due to operational error and eliminate it if necessary).

Calculate the cell proliferation inhibition rate using the following formula (to eliminate blank background interference):Inhibition Rate (%) = [1 - (Average OD Value of Experimental Group - Average OD Value of Blank Control Group) / (Average OD Value of Solvent Control Group/3-MA Control Group/HCQ Control Group - Average OD Value of Blank Control Group)] × 100%

(Note: The TA Alone Group uses the "Solvent Control Group" as the reference; the TA + 3-MA Combination Group uses the "3-MA Control Group" as the reference; the TA + HCQ Combination Group uses the "HCQ Control Group" as the reference.)

2. IC_{50} Calculation and Curve Plotting

Analyze data using GraphPad Prism 9.0 software. Create a "XY Experiment", set the X-axis as "TA Concentration (log-transformed, Log10)" and the Y-axis as "Inhibition Rate (%)".

Select the "Log (inhibitor) vs. response - Variable slope" model to fit the dose–response curve. Set the "Y-axis range to 0-100" and calculate the IC_{50} values (with 95% confidence intervals, CI) for TA alone and TA combined with 3-MA/HCQ at different time points (12 h, 24 h, 48 h).

3. Statistical Analysis

Express data as "mean ± standard deviation (x±s)". Perform at least 3 independent replicate experiments for each group (to avoid single-experiment errors).

Use one-way analysis of variance (One-way ANOVA) followed by Dunnett's multiple comparison test to compare differences between each drug treatment group and the

corresponding control group. A P-value < 0.05 indicates a statistically significant difference, and P < 0.01 indicates a highly significant difference. Mark these differences in graphs with "*" and "**", respectively.
